# Supplementary material for: Factors associated with aspirin resistance in diabetic patients: A metabolic and inflammatory profile analysis
Source: PLoS One. 2025 Sep 11;20(9):e0332323. doi: 10.1371/journal.pone.0332323 (PMC12425219; doi:10.1371/journal.pone.0332323)
Supplement: Attachment — Submitted filename: Response to Reviewers.docx [file pone.0332323.s003.docx]

Dear Editor and Reviewers:

Thanks for your letter and for the reviewers’ comments concerning our manuscript entitled “Factors Influencing Aspirin Resistance in Diabetic Patients: A Metabolic and Inflammatory Profile Analysis” (ID: PONE-D-25-04526). Those comments are all valuable and very helpful for revising and improving our paper, as well as the important guiding significance to our researches. We have studied comments carefully and have made correction which we hope meet with approval. Revised portion are marked in red in the paper. The main corrections in the paper and the responds to the reviewer’s comments are as following:

**The academic editor:**

If applicable, we recommend that you deposit your laboratory protocols in protocols.io to enhance the reproducibility of your results. Protocols.io assigns your protocol its own identifier (DOI) so that it can be cited independently in the future. For instructions see: [https://journals.plos.org/plosone/s/submission-guidelines#](https://track.editorialmanager.com/CL0/https://journals.plos.org/plosone/s/submission-guidelines%23loc-laboratory-protocols/1/010f0195ad1449fa-36c9a2a7-f4d1-4add-bd2b-e8a9c1d2b434-000000/6Fi-zGxlgfI_8OvEC_XXuEZmv2BMHGAy4MCu63eP14I=203)

[loc-laboratory-protocols](https://track.editorialmanager.com/CL0/https://journals.plos.org/plosone/s/submission-guidelines%23loc-laboratory-protocols/1/010f0195ad1449fa-36c9a2a7-f4d1-4add-bd2b-e8a9c1d2b434-000000/6Fi-zGxlgfI_8OvEC_XXuEZmv2BMHGAy4MCu63eP14I=203). Additionally, PLOS ONE offers an option for publishing peer-reviewed Lab Protocol articles, which describe protocols hosted on protocols.io. Read more information on sharing protocols at [https://plos.org/protocols?utm_medium=editorial-email&utm_source=authorletters&utm_campaign=protocols](https://track.editorialmanager.com/CL0/https://plos.org/protocols?utm_medium=editorial-email&utm_source=authorletters&utm_campaign=protocols/1/010f0195ad1449fa-36c9a2a7-f4d1-4add-bd2b-e8a9c1d2b434-000000/5ExDb5gpKH1iC4x1Ya9JIaATS5EoZ1IW0oahCxCADg8=203).

**Response:** Thank you for your suggestion. After checking, the experimental methods involved in my research have been described in detail in the paper, and there are no specific laboratory protocols that need to be uploaded independently. If necessary, I am happy to provide further explanations or make adjustments according to your guidance.

Journal Requirements:

1. When submitting your revision, we need you to address these additional requirements.

Please ensure that your manuscript meets PLOS ONE's style requirements, including those for file naming. The PLOS ONE style templates can be found at

[https://journals.plos.org/plosone/s/file?id=wjVg/PLOSOne_formatting_sample_main_body.pdf](https://journals.plos.org/plosone/s/file?id=wjVg/PLOSOne_formatting_sample_main_body.pdf) and [https://journals.plos.org/plosone/s/file?id=ba62/PLOSOne_formatting_sample_title_authors_affiliations.pdf](https://track.editorialmanager.com/CL0/https://journals.plos.org/plosone/s/file?id=ba62/PLOSOne_formatting_sample_title_authors_affiliations.pdf/1/010f0195ad1449fa-36c9a2a7-f4d1-4add-bd2b-e8a9c1d2b434-000000/JpqpBshQEGULBG8qTLVwjJnxyfb84wbJzKDVw-D2wyQ=203)

**Response:** Thank you very much for your reminder. We have checked it again, and ensured that the manuscript meet PLOS ONE’s style requirements, including those for file naming.

2.We note that your Data Availability Statement is currently as follows: [All relevant data are within the manuscript and its Supporting Information files.]
Please confirm at this time whether or not your submission contains all raw data required to replicate the results of your study. Authors must share the “minimal data set” for their submission. PLOS defines the minimal data set to consist of the data required to replicate all study findings reported in the article, as well as related metadata and methods ([https://journals.plos.org/plosone/s/data-availability#loc-minimal-data-set-definition](https://journals.plos.org/plosone/s/data-availability#loc-minimal-data-set-definition)).
For example, authors should submit the following data:
- The values behind the means, standard deviations and other measures reported;
- The values used to build graphs;
- The points extracted from images for analysis.
Authors do not need to submit their entire data set if only a portion of the data was used in the reported study.
If your submission does not contain these data, please either upload them as Supporting Information files or deposit them to a stable, public repository and provide us with the relevant URLs, DOIs, or accession numbers. For a list of recommended repositories, please see [https://journals.plos.org/plosone/s/recommended-repositories.](https://journals.plos.org/plosone/s/recommended-repositories.)
If there are ethical or legal restrictions on sharing a de-identified data set, please explain them in detail (e.g., data contain potentially sensitive information, data are owned by a third-party organization, etc.) and who has imposed them (e.g., an ethics committee). Please also provide contact information for a data access committee, ethics committee, or other institutional body to which data requests may be sent. If data are owned by a third party, please indicate how others may request data access.

**Response:** Thank you for your feedback. We confirm that our submission fully complies with PLOS's data availability policy.

(1) .Data Availability Statement Update:We have uploaded the complete minimal dataset required to replicate all study findings as a Supporting Information file named "**data**" (format: .xlsx). This file contains:Raw numerical values underlying summary statistics (means, SDs, etc.);Source data for all figures and graphs;Coordinates/extracted points from image analyses;Metadata describing variables, experimental protocols, and data processing steps.
(2) .Accessibility:The dataset is freely accessible alongside the manuscript files. No ethical, legal, or third-party restrictions apply to this de-identified data.
(3) .Compliance Statement:Our updated Data Availability Statement now explicitly references the uploaded file:"All raw data required to replicate the study findings are available in the Supporting Information file 'data'."

Please let us know if any additional clarifications or data formatting adjustments are needed.

Reviewer 1:

1. Blood sample collection data for biochemical analysis is not reported.

**Response:** Thank you for highlighting this omission. We have now added detailed blood sample collection protocols to the Methods section to improve methodological transparency. Below is the revised text:”Blood samples were collected from all participants after an overnight fast (≥8 hours) to minimize diurnal and dietary variability. Venous blood was drawn into EDTA-anticoagulated tubes for plasma separation and serum-separating tubes (SST) for serum biomarkers. Plasma and serum were isolated by centrifugation at 3,000 rpm for 10 minutes within 30 minutes of collection. Aliquots were stored at −80°C until analysis to prevent analyte degradation. Hemolyzed or lipemic samples were excluded based on visual inspection.”(Page [6], Lines [132-138]).

We appreciate your feedback, which has strengthened the methodological rigor of our study.

2.Why did seven-day treatment used what would be the impact if the drug is prescribed for longer duration of time, provide some reference.

**Response:** We thank the reviewer for their thoughtful inquiry regarding our decision to implement a seven-day treatment period for aspirin administration and the potential effects of extending this duration. We provide a comprehensive explanation below, grounded in the biological properties of platelets and supported by relevant literature.

**Rationale for the Seven-Day Treatment Period：**

The selection of a seven-day treatment period with low-dose enteric-coated aspirin (81–100 mg/day) was deliberate and based on established scientific principles. Platelets have a lifespan of approximately 7–10 days, and aspirin exerts its antiplatelet effect by irreversibly inhibiting COX-1, thereby preventing the synthesis of TxA2. This inhibition persists for the lifespan of the affected platelets. A seven-day duration ensures that the majority of circulating platelets are exposed to aspirin’s effect, enabling a reliable evaluation of its cumulative antiplatelet activity. In our study, this was measured through urinary 11dhTxB2 levels following the intervention.

This timeframe aligns with widely accepted research protocols for assessing aspirin’s pharmacodynamic effects. For example:

Eikelboom et al. (2002) employed a minimum of seven days of aspirin administration to evaluate thromboxane biosynthesis in patients at high cardiovascular risk, setting a benchmark for this duration.

Lopez et al. (2014) utilized a seven-day regimen to study platelet thromboxane responses in diabetic patients with coronary artery disease, validating its relevance to our study population.

Parish et al. (2023) reinforced this approach by establishing consensus criteria for classifying aspirin responders based on 11dhTxB2 levels after a seven-day protocol.

These studies collectively affirm that a seven-day treatment period is optimal for investigating aspirin’s short-term impact on platelet function and thromboxane suppression.

**Implications of a Longer-Term Aspirin Regimen：**

Extending aspirin administration beyond seven days—potentially to weeks or months—would likely maintain thromboxane suppression, given aspirin’s irreversible COX-1 inhibition and the continuous turnover of platelets. However, prolonged use could introduce additional considerations, particularly in our study population of diabetic individuals. These include:

Aspirin Resistance: Extended therapy might increase the risk of aspirin resistance in some patients, potentially due to accelerated platelet turnover or alternative thromboxane production pathways (e.g., COX-2 upregulation under hyperglycemic conditions), as noted by Simeone et al. (2018). This could reduce efficacy over time.

Confounding Variables: Longer treatment durations may be affected by factors such as changes in glycemic control, patient compliance, or side effects (e.g., gastrointestinal bleeding), which could obscure the interpretation of thromboxane levels.

In conclusion, the seven-day treatment period was chosen to correspond with the platelet lifespan and established research standards, providing a robust framework for assessing aspirin’s antiplatelet efficacy. While a longer duration might sustain thromboxane suppression, it is unnecessary for our study’s objectives and could introduce confounding factors. We believe this approach is scientifically justified and consistent with prior literature.

**References**

1.Eikelboom JW, Hirsh J, Weitz JI, Johnston M, Yi Q, Yusuf S. Aspirin-resistant thromboxane biosynthesis and the risk of myocardial infarction, stroke, or cardiovascular death in patients at high risk for cardiovascular events. Circulation. 2002;105(14):1650-1655.

2.Lopez LR, Guyer KE, Torre IG, Pitts KR, Matsuura E, Ames PR. Platelet thromboxane (11-dehydro-Thromboxane B2) and aspirin response in patients with diabetes and coronary artery disease. World J Diabetes. 2014;5(2):115-127.

3.Parish S, Buck G, Aung T, Mafham M, Clark S, Hill MR, et al. Effect of low-dose aspirin on urinary 11-dehydro-thromboxane B2 in the ASCEND (A Study of Cardiovascular Events iN Diabetes) randomized controlled trial. Trials. 2023;24(1):166.

4.Simeone P, Boccatonda A, Liani R, Santilli F. Significance of urinary 11-dehydro-thromboxane B(2) in age-related diseases: Focus on atherothrombosis. Ageing Res Rev. 2018;48:51-78.

3.Were the samples collected 24 h after the last aspirin intake?

**Response:** We thank the reviewer for their careful attention to the timing of sample collection. In response to the query, we confirm that urine samples for the measurement of 11dhTxB2 were indeed collected 24 hours after the last aspirin intake. This timing was deliberately chosen to ensure a standardized assessment of aspirin's antiplatelet effect. By collecting samples at this interval, we could evaluate thromboxane suppression at a consistent time point following the final dose. Given that aspirin irreversibly inhibits COX-1, the 24-hour post-dose collection ensures that the observed 11dhTxB2 levels reflect the cumulative impact of the seven-day treatment period.

To make this detail explicit and address the reviewer’s concern, we have updated the revised manuscript with the following clarification in the **Methods** section (Page [7], Lines [148-150]):
"Urine samples for 11dhTxB2 measurement were collected 24 hours after the last aspirin intake to standardize the timing of sample collection relative to the final dose."

We believe this addition enhances the transparency of our methodology and aligns with established practices in studies evaluating aspirin’s pharmacodynamic effects. We appreciate the reviewer’s diligence in raising this point, as it has helped us strengthen the manuscript.

4. Was the drug given in the range of 81-100mg according to body weight or was some specific dose given to patients?

**Response:** Thank you for your query regarding the aspirin dosage in our study. To address your question directly: all patients received a fixed daily dose of 100 mg of low-dose enteric-coated aspirin for seven consecutive days. The dosage was not adjusted based on body weight or any other individual characteristics. Instead, a specific dose of 100 mg per day was administered uniformly to all participants.

To provide further context, the dosage range of 81-100 mg mentioned in the study reflects the commonly accepted therapeutic range for aspirin’s antiplatelet effects. In this case, we selected a fixed dose of 100 mg/day as the standard for all participants, which aligns with typical clinical study designs evaluating aspirin’s inhibition of platelet COX-1 activity in adults. This fixed-dose approach eliminates the need for weight-based adjustments, as the therapeutic effect is achieved consistently within this dosage range across adult populations.

We hope this clarifies the administration protocol used in our study!

5. Provide information about the risk assessment based on levels of 11-dehydro-TXB2.

**Response:**Thank you for your valuable suggestion. We acknowledge the importance of elaborating on the risk assessment associated with levels of urinary 11dhTxB2 in the context of our study. Below, we have provided additional information based on the data presented in our manuscript and supported by relevant literature, which has been integrated into the revised manuscript under the “Discussion” section.

In our study, diabetic patients exhibited significantly higher baseline urinary 11dhTxB2 levels (median: 2324.58 pg/mg creatinine, IQR: 1447.50–3401.08) compared to healthy controls (median: 1452.51 pg/mg creatinine, IQR: 819.70–2302.12; P = 0.001). Post-aspirin intervention, 11dhTxB2 levels remained elevated in the diabetic group (median: 1205.31 pg/mg creatinine, IQR: 877.52–1446.69) compared to controls (median: 1058.02 pg/mg creatinine, IQR: 641.41–1252.66; P = 0.007). These persistently elevated levels suggest a prothrombotic state in diabetic patients, even after aspirin therapy, which aligns with the observed 2.7-fold higher prevalence of biochemical aspirin resistance (20.8% vs. 7.6%, P = 0.036).

The clinical relevance of 11dhTxB2 levels as a risk marker for cardiovascular events has been well-documented. Eikelboom et al. (2002) demonstrated that elevated urinary 11dhTxB2 levels are strongly associated with an increased risk of myocardial infarction, stroke, or cardiovascular death in high-risk patients, independent of traditional risk factors such as hypertension, hyperlipidemia, or smoking [1]. Specifically, their study reported a dose-response relationship, with higher quartiles of 11dhTxB2 correlating with incrementally greater cardiovascular risk (hazard ratio: 1.8 for the highest vs. lowest quartile, P < 0.001). Although our study did not directly assess clinical outcomes, the elevated baseline 11dhTxB2 levels in diabetic patients (60% higher than controls) suggest a heightened thrombotic risk profile, consistent with this prior evidence.

Furthermore, post-aspirin 11dhTxB2 levels provide insight into aspirin’s efficacy in mitigating this risk. Our threshold for biochemical aspirin resistance (≥1500 pg/mg creatinine) aligns with consensus criteria [2], and the 20.8% resistance rate in diabetic patients indicates a substantial subset with inadequate thromboxane suppression. Persistent 11dhTxB2 levels ≥1500 pg/mg creatinine post-therapy have been linked to a 2- to 4-fold increased risk of adverse cardiovascular events in prior studies [3]. For instance, McCullough et al. (2017) found that patients with stable coronary artery disease and elevated 11dhTxB2 had higher all-cause mortality (adjusted HR: 1.67, 95% CI: 1.03–2.71, P = 0.038) [4]. While our study focused on biochemical resistance rather than clinical endpoints, these findings imply that diabetic patients with post-ASA 11dhTxB2 levels ≥1500 pg/mg creatinine (approximately 20.8% of our cohort) may face elevated cardiovascular risk, warranting closer monitoring or alternative therapeutic strategies.

To enhance the risk assessment discussion in our manuscript, we have revised the “**Discussion**” section as follows:

"The elevated baseline urinary 11dhTxB2 levels in diabetic patients (2324.58 vs. 1452.51 pg/mg creatinine in controls, P = 0.001) underscore a heightened thrombotic risk, consistent with prior evidence linking thromboxane biosynthesis to cardiovascular events[14]. Eikelboom et al. (2002) reported that higher 11dhTxB2 levels independently predict myocardial infarction, stroke, or cardiovascular death, with a hazard ratio of 1.8 for the highest versus lowest quartile (P < 0.001) [11]. Post-aspirin, the persistence of elevated 11dhTxB2 (1205.31 vs. 1058.02 pg/mg creatinine in controls, P = 0.007) and a 20.8% prevalence of biochemical resistance (≥1500 pg/mg creatinine) in our diabetic cohort further suggest an attenuated risk reduction. Previous studies indicate that post-ASA 11dhTxB2 levels ≥1500 pg/mg creatinine are associated with a 2- to 4-fold increased risk of adverse cardiovascular outcomes [15], highlighting the potential prognostic utility of this biomarker in identifying high-risk diabetic patients. While our study did not assess clinical events, these findings support the need for personalized risk stratification and therapeutic optimization in this population."

We hope this addition adequately addresses your comment by contextualizing 11dhTxB2 levels within a risk assessment framework, leveraging both our data and established literature. Please let us know if further clarification or adjustments are required.

**References:**

1.Eikelboom JW, Hirsh J, Weitz JI, Johnston M, Yi Q, Yusuf S. Aspirin-resistant thromboxane biosynthesis and the risk of myocardial infarction, stroke, or cardiovascular death in patients at high risk for cardiovascular events. Circulation. 2002;105(14):1650-1655. doi: 10.1161/01.CIR.0000013777.21160.07.

2.Parish S, Buck G, Aung T, Mafham M, Clark S, Hill MR, et al. Effect of low-dose aspirin on urinary 11-dehydro-thromboxane B2 in the ASCEND (A Study of Cardiovascular Events iN Diabetes) randomized controlled trial. Trials. 2023;24(1):166. doi: 10.1186/s13063-023-07198-z.

3.Lopez LR, Guyer KE, Torre IG, Pitts KR, Matsuura E, Ames PR. Platelet thromboxane (11-dehydro-Thromboxane B2) and aspirin response in patients with diabetes and coronary artery disease. World J Diabetes. 2014;5(2):115-127. doi: 10.4239/wjd.v5.i2.115.

4.McCullough PA, Vasudevan A, Sathyamoorthy M, Schussler JM, Velasco CE, Lopez LR, et al. Urinary 11-Dehydro-Thromboxane B(2) and Mortality in Patients With Stable Coronary Artery Disease. Am J Cardiol. 2017;119(7):972-977. doi: 10.1016/j.amjcard.2016.12.004.

6. Was the study involved patients with type1 and type2 diabetes?

**Response:**Thank you for your valuable comment. The study specifically enrolled patients with type 2 diabetes (DM) only. Patients with type 1 diabetes were not included in the study, as the focus was to investigate the factors influencing aspirin resistance in individuals with type 2 diabetes. We have clarified this point in the revised manuscript to ensure the study's population is accurately described(Page [5], Lines [108-110]):“A total of 53 patients with type 2 diabetes (DM) were recruited from the outpatient and inpatient departments of Chaohu Hospital Affiliated to Anhui Medical University.”

7. Consider revising sentence structures, refining the flow of ideas, and ensuring consistency throughout.

**Response:**We sincerely appreciate the reviewer’s valuable feedback and constructive suggestions, which have been instrumental in improving the quality of our manuscript. We have carefully considered your comments and made several adjustments to enhance the manuscript’s readability and clarity. While these revisions are not exhaustive, we believe they address the main concerns raised.

**Sentence Structures:** We have reviewed the manuscript and simplified some complex sentences, particularly in the introduction and discussion sections, to improve overall flow.

**Flow of Ideas:** We have added transitional sentences between sections to help readers better follow the logical progression of the article.

**Consistency:** We have checked the use of terminology and formatting to ensure a uniform style throughout the manuscript.

We believe these adjustments have strengthened the manuscript while preserving its core findings and contributions. We are grateful for the reviewer’s guidance and look forward to any further suggestions.

Special thanks to you for your good comments.

Reviewer 2:
Overall I found the manuscript to be informative and well-written. My comments are minor, but I feel resolution would lend greater clarity to the message.
ABSTRACT: The abstract is concise and reflective of the manuscript.
**Response**:Thank you for your positive feedback on the abstract. We greatly appreciate your recognition of its conciseness and its ability to accurately reflect the manuscript's content. The abstract is a critical component of our work, serving as a gateway for readers to quickly grasp the study's key findings and significance. We invested considerable effort in crafting it to ensure it effectively highlights the novelty and importance of our research, aiming to engage readers from the outset.

INTRODUCTION: The introduction is clear and informative.
**Response**:Thank you for your positive feedback on the introduction. We are delighted to hear that you found it clear and informative. The introduction plays a vital role in our manuscript by providing essential background and motivation for the study. We put considerable effort into ensuring it effectively conveys the significance and novelty of our research, particularly with regard to the metabolic and inflammatory factors contributing to aspirin resistance in diabetic patients.

As outlined in the introduction, our study offers fresh insights that could improve cardiovascular disease prevention strategies for this high-risk group.

We truly value your feedback and would greatly appreciate any additional suggestions you might have for the other sections of the manuscript.

METHODS: The authors state this is a 'prospective case-control design.' Technically, a case-control design is, by definition, a retrospective study in which 'cases' are patients with the outcome in question while 'controls' are patients without the outcome. The purpose of these studies is to define the exposures most associated with the outcome, with the presumption that these exposures are implicated in the cause. The current study is more of a matched prospective cohort design, as patients do or do not have an exposure of interest (diabetes), and they are being evaluated for an outcome of interest (aspirin resistance). There is a question about matching as noted below.

**Response**:We sincerely thank the reviewer for their insightful comment and for drawing attention to the classification of our study design. We appreciate the opportunity to address this point and improve the clarity of our manuscript.

The reviewer is correct in noting that a case-control design is, by definition, a retrospective study where 'cases' are individuals with the outcome of interest, and 'controls' are those without it, with the aim of identifying exposures associated with that outcome. In our manuscript, we described the study as a "prospective case-control design," but upon reflection and in light of the reviewer’s feedback, we acknowledge that this terminology does not accurately reflect our methodology.

In this study, we enrolled diabetic patients (the exposed group) and healthy controls (the unexposed group), and then prospectively evaluated both groups for the outcome of interest—aspirin resistance—following a 7-day intervention with low-dose aspirin. This approach aligns with a prospective cohort study, where participants are defined by their exposure status (diabetes versus no diabetes) at the outset and followed forward in time to assess the incidence of the outcome (aspirin resistance). Furthermore, the reviewer’s observation that this is a "matched prospective cohort design" is apt, as we matched the healthy controls to the diabetic patients based on age and sex to control for potential confounding by these variables.

To address the reviewer’s comment, we have taken the following steps:

**Clarification of Study Design:** We have revised the manuscript to accurately describe the study as a prospective cohort study with matching. This change eliminates the misleading "case-control" terminology and better reflects the prospective nature of our data collection and analysis.

**In the Methods section, we now state:**

"This study employed a prospective cohort design with matching. A total of 53 patients with type 2 diabetes (DM) were recruited from the outpatient and inpatient departments of Chaohu Hospital Affiliated to Anhui Medical University. All participants were diagnosed according to internationally recognized diagnostic criteria for diabetes. The control group consisted of 66 age- and sex-matched healthy individuals, who were selected from hospital staff and local community members, all of whom had normal blood glucose levels."(Page [5], Lines [108-114]).

These revisions ensure that the study design is accurately represented and that the matching process is transparently described. We believe this addresses the reviewer’s concerns and enhances the scientific precision of our manuscript. We are grateful for the reviewer’s expertise and guidance, which have been instrumental in refining our work.

It would be helpful to know if these subjects have Type 1 DM or Type 2 DM. The BMI suggests Type 1 DM; however, this may not be the case. This is important because of the proposed mechanism (insulin resistance) is considered to be the underlying pathobiology in Type 2 but not Type 1 DM (though itt can exist in both). Also, given the age of 62 in the DM group, since Type 1 DM is often a disease of onset in the childhood or teenage years, the duration of exposure to the disease would be substantially longer in those with Type 1 DM vs those with Type 2, which often has onset in adulthood.

**Response**:Thank you for your valuable feedback on our manuscript. We appreciate the opportunity to address your concerns and clarify key aspects of our study.

**Clarification on Diabetes Type**

You raised an important question regarding whether the subjects in this study have Type 1 or Type 2 DM, particularly noting that the reported BMI might suggest Type 1 DM. We would like to clarify that this study exclusively enrolled patients with Type 2 DM. Patients with Type 1 diabetes were not included, as our research focuses on factors influencing aspirin resistance specifically in individuals with Type 2 DM. This point was also addressed in our response to Reviewer 1 and has been explicitly stated in the revised manuscript (Page [5], Lines [108-110]):“A total of 53 patients with type 2 diabetes (DM) were recruited from the outpatient and inpatient departments of Chaohu Hospital Affiliated to Anhui Medical University.”

The mechanism of insulin resistance, which is central to our study, is predominantly associated with Type 2 DM, although it may occur in both types to varying degrees. We hope this clarification resolves any ambiguity regarding the study population.

**Addressing BMI Concerns**

We acknowledge your observation that the BMI values in our cohort may appear lower than those typically expected in some populations with Type 2 DM. All participants were diagnosed with Type 2 DM based on internationally recognized criteria, including:Fasting plasma glucose (FPG) ≥7.0 mmol/L,2-hour postprandial blood glucose ≥11.1 mmol/L during an oral glucose tolerance test (OGTT), or Glycated hemoglobin (HbA1c) ≥6.5%.

The relatively lower BMI in our sample is likely attributable to regional and ethnic differences in body composition and diabetes phenotype. Specifically, research has demonstrated that Asian populations, including Chinese individuals, can develop Type 2 DM at lower BMI levels compared to Western populations. For example, Chan et al. (2009) highlight that the risk of Type 2 DM increases at a lower BMI threshold in Asian cohorts [1]. This characteristic is consistent with our study population, which comprises Chinese patients, and explains the observed BMI values.

You also noted the median age of the DM group (62 years) and its potential implications, given that Type 1 DM typically has an earlier onset. The median age of 62 aligns with the epidemiology of Type 2 DM, which commonly manifests in adulthood.

We sincerely thank you for your insightful comments, which have helped us improve the clarity and rigor of our study. We hope these clarifications adequately address your concerns. Please let us know if further adjustments are needed.

**Reference**

1.Chan JC, Malik V, Jia W, Kadowaki T, Yajnik CS, Yoon KH, Hu FB. Diabetes in Asia: epidemiology, risk factors, and pathophysiology. JAMA. 2009 May 27;301(20):2129-40. doi: 10.1001/jama.2009.726. PMID: 19470990.

As far as the outcome, the authors defined it as a specific threshold of 11dhTxB2 level of <1500 pg/mg. They give a reference that this threshold is in accordance with consensus criteria, but this does not seem to be entirely accurate. The reference cited is that of a cutoff value for a specific assay as found in the FDA submission ([https://www.accessdata.fda.gov/cdrh_docs/reviews/K062025.pdf](https://www.accessdata.fda.gov/cdrh_docs/reviews/K062025.pdf)). This may be appropriate, but more clarification on why this specific value is used would be helpful - has this specific threshold been associated with specific outcomes?
**Response**:Thank you for your insightful comment regarding the threshold used to define aspirin resistance in our study. We appreciate the opportunity to clarify the rationale behind selecting 1500 pg/mg creatinine of urinary 11dhTxB2 as the cutoff for distinguishing between aspirin responders and non-responders.

You correctly pointed out that the reference we cited ([https://www.accessdata.fda.gov/cdrh_docs/reviews/K062025.pdf](https://www.accessdata.fda.gov/cdrh_docs/reviews/K062025.pdf)) pertains to an FDA submission for a specific assay, rather than a broad consensus criterion for defining aspirin resistance. We acknowledge that our initial phrasing may have implied a wider acceptance of this threshold than is supported by the literature, and we apologize for any lack of clarity. The 1500 pg/mg creatinine threshold is indeed tied to the validation data of the specific assay used in our study, and we welcome the chance to elaborate on why this value was chosen and its relevance to our research.

**Rationale for Choosing the 1500 pg/mg creatinine Threshold**

In our study, we employed urinary 11dhTxB2 as a biomarker to assess platelet activation and the efficacy of aspirin in suppressing thromboxane biosynthesis. The threshold of 1500 pg/mg creatinine was selected based on the assay’s validation data, as documented in the FDA submission, which established this cutoff to differentiate between aspirin responders (levels <1500 pg/mg creatinine) and non-responders (levels ≥1500 pg/mg creatinine). This value has been utilized in prior research with the same assay to categorize biochemical aspirin resistance, which reflects persistent thromboxane production despite aspirin therapy.

We recognize that aspirin resistance is a complex and poorly standardized concept. Different studies have employed various methods—such as platelet aggregation tests or thromboxane metabolite measurements—and have proposed different thresholds depending on the assay or population studied. For our study, the choice of 1500 pg/mg creatinine was guided by the assay’s characteristics and its established use in similar research contexts, ensuring consistency with the biochemical framework we aimed to investigate.

**Association with Clinical Outcomes**

You raised an important question about whether this specific threshold has been directly associated with clinical outcomes. While the exact cutoff of 1500 pg/mg creatinine has not been universally validated against clinical endpoints in large-scale trials, elevated urinary 11dhTxB2 levels (above varying thresholds) have been consistently linked to increased cardiovascular risk in the literature. For example:

Eikelboom et al. (2002): This study found that patients in the highest quartile of urinary 11dhTxB2 levels had a significantly greater risk of myocardial infarction, stroke, or cardiovascular death compared to those in the lowest quartile (hazard ratio 1.8, P < 0.001)[1]. Although this study did not use the precise 1500 pg/mg creatinine threshold, it supports the broader principle that incomplete thromboxane suppression is associated with adverse outcomes.

Other Supporting Evidence: Additional studies, such as Lopez et al. (2014) and Parish et al. (2023), have shown that persistent elevation of urinary 11dhTxB2 after aspirin administration correlates with a higher incidence of cardiovascular events, particularly in high-risk populations like those with diabetes or coronary artery disease[2,3].

While these studies do not specifically validate the 1500 pg/mg creatinine cutoff, they reinforce the clinical relevance of using urinary 11dhTxB2 as a marker of aspirin resistance and its association with poorer prognosis when thromboxane levels remain elevated.

**Application in Our Study**

The primary objective of our study was to investigate biochemical aspirin resistance as a marker of platelet hyperreactivity in diabetic patients, rather than to directly correlate the 1500 pg/mg creatinine threshold with clinical events. We used this cutoff to categorize participants into aspirin responders and non-responders, enabling us to explore differences in metabolic and inflammatory profiles between these groups. Our results showed that diabetic patients with 11dhTxB2 levels ≥1500 pg/mg creatinine exhibited significantly higher BMI, HOMA-IR, and hs-CRP levels compared to those with levels <1500 pg/mg creatinine. These findings suggest that this threshold effectively identifies a subgroup with heightened metabolic-inflammatory dysregulation, which may contribute to cardiovascular risk indirectly.

To address your concern and improve transparency, we have revised the manuscript (Page [8], Lines [163-176]) to clarify the basis for the threshold and acknowledge its limitations:

"The threshold of 1500 pg/mg creatinine for urinary 11dhTxB2 was selected based on the validation data for the specific assay used in this study, as detailed in the FDA submission[11].Participants were classified as good ASA responders (post-administration urinary 11dhTxB2 < 1500 pg/mg creatinine) or poor ASA responders (biochemical ASA resistance) (≥ 1500 pg/mg creatinine). This classification reflects impaired COX-1-mediated TxA2 suppression, a key mechanism underlying aspirin’s antiplatelet efficacy. The study specifically investigates biochemical ASA resistance, defined as persistent thromboxane biosynthesis despite therapy, and its association with metabolic dysregulation in DM. While this cutoff has been employed in previous research to distinguish aspirin responders from non-responders, we acknowledge that it is assay-specific and not a universal consensus criterion. Although direct associations between this exact threshold and clinical outcomes are limited, elevated urinary 11dhTxB2 levels have been consistently linked to increased cardiovascular risk in multiple studies [11,15,16]."

In summary, the 1500 pg/mg creatinine threshold was chosen for its alignment with the assay’s validation and prior research usage, rather than as a universally validated clinical predictor. While it may not have a direct, assay-independent link to specific outcomes, the broader evidence supports the use of urinary 11dhTxB2 as a meaningful indicator of aspirin response and cardiovascular risk. We hope this explanation addresses your query and justifies our methodological choice. Thank you again for your thoughtful feedback, which has undoubtedly strengthened our manuscript.

**References**

1.Eikelboom JW, Hirsh J, Weitz JI, Johnston M, Yi Q, Yusuf S. Aspirin-resistant thromboxane biosynthesis and the risk of myocardial infarction, stroke, or cardiovascular death in patients at high risk for cardiovascular events. Circulation. 2002;105(14):1650-1655. doi:10.1161/01.CIR.0000013777.21160.07.

2.Lopez LR, Guyer KE, Torre IG, Pitts KR, Matsuura E, Ames PR. Platelet thromboxane (11-dehydro-Thromboxane B2) and aspirin response in patients with diabetes and coronary artery disease. World J Diabetes. 2014;5(2):115-127. doi:10.4239/wjd.v5.i2.115.

3.Parish S, Buck G, Aung T, Mafham M, Clark S, Hill MR, et al. Effect of low-dose aspirin on urinary 11-dehydro-thromboxane B2 in the ASCEND (A Study of Cardiovascular Events iN Diabetes) randomized controlled trial. Trials. 2023;24(1):166. doi:10.1186/s13063-023-07198-z.

A brief desciption of recruitment methods would be helpful to understand how these patients were identified and from what population they were selected.

**Response**:Thank you for your valuable feedback. We have revised the manuscript to clarify the recruitment methods as follows.

Revised Text in Methods Section (**Study Design and Participants**)(Page [5], Lines [108-114]):”This study employed a prospective cohort design with matching. A total of 53 patients with type 2 diabetes (DM) were recruited from the outpatient and inpatient departments of Chaohu Hospital Affiliated to Anhui Medical University. All participants were diagnosed according to internationally recognized diagnostic criteria for diabetes. The control group consisted of 66 age- and sex-matched healthy individuals, who were selected from hospital staff and local community members, all of whom had normal blood glucose levels. ”

We sincerely appreciate your constructive suggestion, which has helped us improve the methodological transparency of the study. Please don't hesitate to contact us if further clarification regarding the recruitment process is required.

For statistical methods, given that this is a repeated measure (each patient contributed two values, one pre- and one post- exposure), an analysis of repeated measures would be appropriate. In its current form, the entire populations are measured with pre- and post-values; however, the change per individual may be more appropriate to identfiy a group of patients in whom aspirin has little effect. For example, are those who do not achieve the pre-defined threshold of 1500 pg/mL those who started well above this threshold, or are there some patients who have little to no response at all? This may signifcantly impact the results and the discussion.
**Response**:Thank you for your valuable suggestions regarding our statistical methods and analytical approach. We fully agree that a more detailed individual-level change analysis, given the repeated-measures nature of the data (pre- and post-medication 11dhTxB2 levels for each patient), could enhance the accuracy and depth of our results. Your recommendation to focus on individual-level responses and identify aspirin non-responders was particularly valuable, significantly enriching our results and discussion.

In response to your comments, we have made the following modifications to the manuscript:

**Repeated-measures analysis:** We employed the Wilcoxon signed-rank test, considering the non-normal distribution of 11dhTxB2 levels, to evaluate individual changes before and after medication. This provides a more robust evaluation of aspirin effects.

**Individual change analysis:** We calculated Δ11dhTxB2 for each patient and analyzed its distribution, revealing inter-individual variability in responses.

**Non-responder characterization:** Following your suggestion, we identified patients with post-treatment 11dhTxB2 ≥1500 pg/mg creatinine. Analysis of their baseline levels and magnitude of change suggested potential genuine aspirin resistance in some patients.

**Manuscript updates:** We have revised the manuscript to clarify the recruitment methods as follows:

Revised Text in "**Statistical Methods**" section(Page [9], Lines [188-189]):”To evaluate within-subject changes in Δ11dhTxB2 levels, we utilized the Wilcoxon signed-rank test.”

Added detailed descriptions of Δ11dhTxB2 distribution and non-responder characteristics in "**Results**"(Page [15], Lines [279-292]):”Biochemical aspirin resistance and response variability in DM patients

The Wilcoxon signed-rank test demonstrated a statistically significant decrease in urinary 11dhTxB2 levels following aspirin administration (Z = -6.123, P < 0.001). The median change in 11dhTxB2 levels (Δ11dhTxB2 = pre-aspirin – post-aspirin) was 1119.27 pg/mg creatinine, with an interquartile range (IQR) of 800.00–1500.00 pg/mg creatinine, reflecting considerable inter-individual variability in aspirin response. Of the 53 diabetic patients, 11 (20.8%) exhibited post-aspirin 11dhTxB2 levels ≥1500 pg/mg creatinine and were classified as aspirin-resistant. This subgroup had a median baseline 11dhTxB2 level of 4500 pg/mg creatinine(IQR: 3200–6800 pg/mg creatinine), significantly higher than the overall cohort median of 2324.58 pg/mg creatinine. Notably, a subset of three patients displayed minimal reduction (Δ11dhTxB2 < 10%) despite baseline levels ranging from 1800–2500 pg/mg creatinine.”

Expanded discussions on aspirin resistance mechanisms and clinical implications in the "**Discussion**" section(Page [21], Lines [408-422]):”Heterogeneity in aspirin response among patients with diabetes

Despite a greater median reduction in 11dhTxB2 levels in the DM group (1119.27 pg/mg creatinine) compared to controls (394.49 pg/mg creatinine), fewer DM patients achieved the threshold of <1500 pg/mg (79.2% vs. 92.4%). This is likely due to significantly higher baseline 11dhTxB2 levels in the DM group (median: 2324.58 pg/mg creatinine) requiring a larger absolute reduction to fall below the threshold.

Our findings highlight heterogeneity in aspirin response among diabetic patients, with a small subgroup showing minimal reduction in 11dhTxB2 levels despite moderate baseline values. This observation points to the existence of true aspirin resistance, potentially attributable to mechanisms such as accelerated platelet turnover or non-COX-1-mediated thromboxane production, consistent with previous reports [6,15]. These results emphasize the importance of tailoring aspirin therapy, particularly for high-risk diabetic patients with elevated baseline thromboxane levels or suboptimal responses to standard doses.”

We believe these revisions adequately address your concerns while substantially improving manuscript quality. Specific changes have been highlighted in the revised manuscript, and we hope you find the updated analyses satisfactory. We reiterate our gratitude for your constructive feedback, which has enabled significant improvements to our work.

RESULTS:
In the results, the authors mention that there is not difference between age and gender distribution. Elsewhere, it is mentioned that this is an age/sex-matched cohort. If this is indeed 'matched,' then the process of matching should be disclosed. If there was no intentional matching during the enrollment period, then this is not the appropriate terminology.

**Response**:Thank you for your thorough review and insightful feedback on our manuscript. We greatly appreciate your attention to the description of our study cohort, particularly regarding the age and gender distribution and the use of the term "age/sex-matched cohort." In response to your comment, we have revised the manuscript to enhance clarity and address your concerns.

Specifically, we have updated the "**Study design and participants**" section as follows(Page [5], Lines [108-114]):
"This study employed a prospective cohort design with matching. A total of 53 patients with type 2 diabetes (DM) were recruited from the outpatient and inpatient departments of Chaohu Hospital Affiliated to Anhui Medical University. All participants were diagnosed according to internationally recognized diagnostic criteria for diabetes. The control group consisted of 66 age- and sex-matched healthy individuals, who were selected from hospital staff and local community members, all of whom had normal blood glucose levels."

This revision confirms that the control group was intentionally selected to match the age and sex distribution of the diabetic group. To further clarify the matching process in response to your query, we would like to explain that healthy individuals were chosen to ensure that the overall age and sex characteristics were comparable between the two groups. This was achieved by selecting controls such that the mean age and the proportion of males and females were similar to those of the diabetic patients. As reported in the results section, statistical analysis confirmed no significant differences in age and gender distribution between the diabetic patients and the control group (P > 0.05), validating the effectiveness of this matching approach.

We believe that these revisions and the accompanying explanation adequately address your comment by confirming the intentional matching and providing insight into how it was implemented, thereby justifying the use of the "age- and sex-matched" terminology. We hope this enhances the transparency and precision of our study design description.

Thank you once again for your valuable input, which has undoubtedly improved the quality of our manuscript.

As noted above, a analysis for repeated measures would be helpful. If available, inclusion of glycosylated hemoglobin measurements may be helpful, and this may be a contributing factor to ASA resistance. The DM group had a more substantial reduction in the outcome measure but fewer achieved the predefined threshold. A better representation of this would be helpful, as noted above, to see exactly in whom this threshold was not acheived. Were higher baseline 11dhTxB2 levels evaluated as a predictor of who did or did not achieve this threshold?

1. **Reviewer Comment:** As noted above, an analysis for repeated measures would be helpful.

**Response**:We thank the reviewer for this valuable suggestion. To address this, we have incorporated a repeated measures analysis using the Wilcoxon signed-rank test to evaluate the within-subject changes in urinary 11-dehydrothromboxane B2 (11dhTxB2) levels before and after aspirin administration in the diabetic (DM) group. This non-parametric test was chosen due to the non-normal distribution of the data, as confirmed by the Shapiro-Wilk test (P < 0.05). The results, now included in the revised manuscript under the "Biochemical aspirin resistance and response variability in DM patients" subsection of the Results section(Page [15], Lines [279-280]), show a significant reduction in 11dhTxB2 levels post-aspirin (Z = -6.123, P < 0.001), with a median change (Δ11dhTxB2) of 1119.27 pg/mg creatinine (IQR: 800.00–1500.00 pg/mg creatinine). This analysis strengthens our assessment of aspirin’s effect over time within the DM group.

2.**Reviewer Comment:** If available, inclusion of glycosylated hemoglobin measurements may be helpful, and this may be a contributing factor to ASA resistance.

**Response**:We appreciate the reviewer’s suggestion to include glycosylated hemoglobin (HbA1c) measurements as a potential contributing factor to ASA resistance. Unfortunately, HbA1c was not measured in this study, as it was not included in the original study design, which focused primarily on metabolic and inflammatory markers such as BMI, hs-CRP, and HOMA-IR, alongside urinary 11dhTxB2 levels. We acknowledge the relevance of HbA1c in the context of ASA resistance and have added a statement in the Limitations section of the revised manuscript to address this(Page [22], Lines [440-442]): “ Finally, the absence of HbA1c measurements limits our ability to assess its potential role in ASA resistance, an important consideration for future studies.” We plan to incorporate HbA1c in subsequent research to explore its impact more comprehensively.

3.**Reviewer Comment:** The DM group had a more substantial reduction in the outcome measure but fewer achieved the predefined threshold. A better representation of this would be helpful, as noted above, to see exactly in whom this threshold was not achieved.

**Response**:We thank the reviewer for highlighting this observation and agree that a clearer representation of this finding would enhance the manuscript. In the DM group, aspirin reduced 11dhTxB2 levels by a median of 1119.27 pg/mg creatinine (41.39%), yet only 42 out of 53 patients (79.2%) achieved the predefined threshold of <1500 pg/mg post-aspirin, compared to a higher proportion in controls (61/66, 92.4%). To address this, we have added the Results section under the revised manuscript(Page [15], Lines [286-292])” Of the 53 diabetic patients, 11 (20.8%) exhibited post-aspirin 11dhTxB2 levels ≥1500 pg/mg creatinine and were classified as aspirin-resistant. This subgroup had a median baseline 11dhTxB2 level of 4500 pg/mg creatinine(IQR: 3200–6800 pg/mg creatinine), significantly higher than the overall cohort median of 2324.58 pg/mg creatinine. Notably, a subset of three patients displayed minimal reduction (Δ11dhTxB2 < 10%) despite baseline levels ranging from 1800–2500 pg/mg creatinine.”Additionally, we have expanded the Discussion section under “Heterogeneity in Aspirin Response Among Patients with Diabetes” to explain this paradox(Page [21], Lines [410-414]): “Despite a greater median reduction in 11dhTxB2 levels in the DM group (1119.27 pg/mg creatinine) compared to controls (394.49 pg/mg creatinine), fewer DM patients achieved the threshold of <1500 pg/mg (79.2% vs. 92.4%). This is likely due to significantly higher baseline 11dhTxB2 levels in the DM group (median: 2324.58 pg/mg creatinine) requiring a larger absolute reduction to fall below the threshold.”

4.**Reviewer Comment**: Were higher baseline 11dhTxB2 levels evaluated as a predictor of who did or did not achieve this threshold?

**Response**:Thank you for your valuable feedback. In response to your question, “Were higher baseline 11dhTxB2 levels evaluated as a predictor of who did or did not achieve this threshold?” we have incorporated additional analyses in the revised manuscript. Specifically, we added a univariate analysis in the “Univariate analysis of aspirin resistance-related factors in DM patients” section, showing significantly higher baseline 11dhTxB2 levels in the ASA-resistant group (4719.54 vs. 2053.08 pg/mg creatinine, P = 0.000). Additionally, a new subsection, “Evaluating Baseline 11dhTxB2 Levels as a Predictive Biomarker for Aspirin Resistance in Diabetic Patients,” was included, where logistic regression analysis revealed that each unit increase in baseline 11dhTxB2 was associated with a 0.2% increase in the odds of aspirin resistance (OR: 1.002, 95% CI: 1.001–1.003, P = 0.002). These updates, reflected in Tables 2 and 4, address your concern and enhance the manuscript’s comprehensiveness. We have expanded the Discussion section with an additional subsection delineating the prognostic implications of baseline 11dhTxB2 levels as a predictive biomarker for aspirin resistance(Page [19], Lines [374-382]):”Furthermore, our study identified baseline 11dhTxB2 levels as a significant predictor of aspirin resistance in diabetic patients. The univariate analysis showed markedly higher baseline levels in the ASA-resistant subgroup (4719.54 vs. 2053.08 pg/mg creatinine, P = 0.000), and logistic regression confirmed that each unit increase in baseline 11dhTxB2 was associated with a 0.2% higher odds of resistance (OR: 1.002, 95% CI: 1.001–1.003, P = 0.002). This suggests that elevated thromboxane biosynthesis at baseline may reflect a prothrombotic state less responsive to ASA, potentially driven by accelerated platelet turnover or non-COX-1 pathways, warranting further mechanistic exploration.”

We thank the reviewers for their critical insights. Implementing repeated measures analysis and evaluating baseline 11dhTxB2 as a resistance predictor strengthened methodological rigor, while clarifying threshold attainment patterns provided clinically actionable findings. These refinements significantly enhanced the study's impact.

The BMI is quite low, and the 75th percentile is well under the threshold typically used to define obesity of 30 kg/m2. Obesity is mentioned as a risk for aspirin resistance; however, there seems to be a relatively low prevalence in this cohort. Perhaps increasing BMI, but not obesity, is the risk?

**Response**:Thank you for your insightful comments on our manuscript. We appreciate your observation regarding the relatively low BMI in our cohort and its implications for the relationship between obesity and aspirin resistance.

In our study, the 75th percentile for BMI in the diabetic group was 26.64 kg/m², which, as you correctly noted, falls below the standard obesity threshold of 30 kg/m². This reflects the characteristics of our study population, which may differ from Western cohorts where higher BMI values are more prevalent. Despite this, our univariate analysis revealed a significant association between higher BMI and aspirin resistance (P = 0.035), suggesting that even within this lower BMI range, increases in BMI may contribute to reduced aspirin efficacy.

We acknowledge that the term "obesity" might be misleading in this context given the cohort’s BMI distribution. To address your concern, we have revised the manuscript to clarify that our findings pertain to the impact of increasing BMI, rather than obesity perse, on aspirin resistance. Specifically, we have adjusted the language in the Results and Discussion sections to emphasize BMI as a continuous variable rather than categorically defining it as obesity. Additionally, in the multivariable linear regression analysis, BMI was identified as an independent predictor of attenuated thromboxane suppression (Δ11dhTxB2, B = 204.9, P = 0.021), further supporting the notion that even modest increases in BMI can influence aspirin response in diabetic patients.	We believe these revisions address your concerns while preserving the integrity of our findings. Thank you for helping us enhance the clarity and precision of our manuscript.

Within the results, there are many instances of interpretation. This should be saved for the discussion, and results should be purely objective and without interpretation. For example, line 239-240 "suggesting that these variables may not independently affect ASA resistance" could be seen as interpretive.

**Response**:Thank you for your valuable feedback on our manuscript. We appreciate your careful review and constructive comments. Regarding your concern about the presence of interpretive statements in the results section, we acknowledge that some phrases, such as "suggesting that these variables may not independently affect ASA resistance," could be perceived as interpretive rather than purely objective.

In response to your comment, we have revised the manuscript to ensure that the results section remains strictly objective, presenting only the data and statistical findings without interpretation.Specifically, we have removed or rephrased statements in the results section that could be seen as interpretive.

We believe these changes address your concern and enhance the clarity and structure of the manuscript. Thank you again for your insightful feedback, which has greatly helped us refine our work.

DISCUSSION:
Also, there is mention of hs-CRP as a 'primary driver.' This is a bold statement - there may be an association, but it is difficult to say this is a driver. If it is a 'driver,' then the presumption is that reducing this level would, in turn, result in improving the outcome in question. That is highly speculative. Also note that hs-CRP is an assay, not a molecule.
**Response**:Thank you for your thoughtful and insightful comments on our manuscript. We greatly appreciate your expertise and the opportunity to refine our work based on your feedback.

We acknowledge your concern regarding the use of "primary driver" to describe hs-CRP. Upon reflection, we agree that this phrasing may imply a causal relationship that our study design—a prospective case-control analysis—does not conclusively establish. Our data demonstrate a robust statistical association between elevated hs-CRP levels and attenuated thromboxane suppression (Δ11dhTxB2), but we recognize that causation remains to be proven. To address this, we have revised the manuscript to use more conservative language, such as "strongly associated with" or "a significant predictor of," which better reflects the scope of our findings.

Furthermore, we appreciate your clarification that hs-CRP is an assay rather than a molecule. To ensure technical accuracy, we have revised the text to consistently describe hs-CRP as a measure of high-sensitivity C-reactive protein, a marker of systemic inflammation, and have specified its role as an assay in the Methods section.

We believe these revisions address your concerns while enhancing the precision and clarity of our manuscript. Thank you again for your valuable input, which has undoubtedly improved our work.

I feel that with the above analyses included, especially an assessment of DM control such as HbA1c, may be informative (for example, are those with poor control more likely to have an attenuated response) and an analysis of repeatead measures, may inform the discussion. Also, acknowleding the limitation of the arbitrary cuttoff for ASA resistance may be important. It seems unlikely that there is no benefit in substantial relative reduction if, indeed, the measure is correlated with clinical outcomes.

**Response**:We sincerely thank the reviewer for their thoughtful and insightful comments, which have greatly helped us refine and strengthen our manuscript. Below, we address each point raised and detail the corresponding revisions made:

**1.Inclusion of HbA1c Analysis**

We appreciate the reviewer’s suggestion to include an assessment of diabetes control, such as HbA1c, to explore its potential role in ASA resistance. Unfortunately, HbA1c was not measured in this study, as it was not part of the original study design, which focused on metabolic-inflammatory markers (e.g., BMI, hs-CRP, HOMA-IR) and urinary 11dhTxB2 levels. We recognize the value of HbA1c in this context and have added a statement in the Limitations section of the revised manuscript(Page [22], Lines [441-442]):”Finally, the absence of HbA1c measurements limits our ability to assess its potential role in ASA resistance, an important consideration for future studies.”

**2.Repeated Measures Analysis**

Following the reviewer’s suggestion, we have already incorporated a repeated measures analysis in response to prior comments, using the Wilcoxon signed-rank test to evaluate within-subject changes in urinary 11dhTxB2 levels before and after ASA administration in the DM group. The results, previously added to the Results section under the subsection "Biochemical Aspirin Resistance and Response Variability in Diabetic Patients", demonstrate a significant reduction in 11dhTxB2 levels post-ASA (Z = -6.123, P < 0.001), with a median change (Δ11dhTxB2) of 1119.27 pg/mg creatinine (IQR: 800.00–1500.00 pg/mg creatinine). This analysis enhances our understanding of ASA’s effect over time in DM patients and informs the discussion as suggested.

**3.Acknowledgment of Arbitrary Cutoff for ASA Resistance**

We agree with the reviewer that the arbitrary cut off for ASA resistance (urinary 11dhTxB2 ≥1500 pg/mg creatinine) has limitations and may not fully capture the clinical implications of ASA’s effect. To address this, we have added the following statement to the Limitations section(Page [23], Lines [447-452]):

“Regarding methodology, the use of an arbitrary cutoff for ASA resistance (urinary 11dhTxB2 ≥1500 pg/mg creatinine) may not fully reflect the clinical benefit of aspirin, particularly in patients who exhibit substantial relative reductions in 11dhTxB2 levels. To address this limitation, future studies should consider both absolute and relative changes in 11dhTxB2 to better elucidate the relationship between biochemical markers and clinical outcomes.”
This revision acknowledges the limitation and aligns with the reviewer’s concern.

**4.Discussion of Relative Reduction in 11dhTxB2**

We appreciate the reviewer’s point that substantial relative reductions in 11dhTxB2, even in ASA-resistant patients, may still confer clinical benefits, especially if the measure correlates with clinical outcomes. To address this, we have expanded the Discussion section by adding a new paragraph under the subsection "The effect of aspirin on urinary 11dhTxB2 levels"(Page [18], Lines [346-352]):”It is noteworthy that,, even in patients classified as ASA-resistant based on the arbitrary cutoff (11dhTxB2 ≥1500 pg/mg creatinine), a substantial relative reduction in 11dhTxB2 levels—such as the 41.39% decrease observed in the DM group—may still indicate clinical benefit. Previous studies have demonstrated that urinary 11dhTxB2 levels correlate with cardiovascular outcomes [11], suggesting that any reduction in thromboxane biosynthesis could mitigate thrombotic risk. Thus, the clinical implications of ASA resistance should be interpreted cautiously.”

We believe these revisions address the reviewer’s concerns comprehensively, enhancing the manuscript’s clarity and scientific rigor. We are grateful for the opportunity to improve our work based on your valuable feedback.

Reviewer 3:

This study examines the factors contributing to aspirin resistance in patients with diabetes mellitus, aiming to enhance cardiovascular disease prevention strategies for this population. It investigates the relationship between urinary 11-dehydro-thromboxane B2 (11dhTxB2) levels and metabolic parameters in diabetic patients to better understand the mechanisms behind inadequate aspirin response.

The findings indicate that diabetes patients exhibit heightened thromboxane production, as evidenced by elevated urinary 11dhTxB2 levels, and a significantly higher prevalence of biochemical aspirin resistance compared to healthy individuals. Increased body mass index (BMI), low-density lipoprotein cholesterol (LDL-C), cystatin-C, high-sensitivity C-reactive protein (hs-CRP), and homeostatic model assessment for insulin resistance (HOMA-IR) suggest that obesity and systemic inflammation are key factors modulating reduced aspirin efficacy. These results imply that platelet activation pathways beyond COX-1 inhibition are involved, highlighting the complexity of aspirin resistance in diabetic patients.

Major remarks

Contrary to the hypothesis, aspirin (ASA) administration reduced 11-dehydro-thromboxane B2 (11dhTxB2) levels in diabetes mellitus (DM) patients compared to controls, indicating greater thromboxane suppression in DM, the intergroup difference did not reach statistical significance. This lack of significance may be attributed to the limited sample size. The study suggested that persistent thromboxane overproduction could be due to hyperglycemia-driven COX-2 upregulation. However, further clarification was not provided. To support this hypothesis, additional analysis of inflammatory or oxidative stress markers could be beneficial.

Univariate analysis showed that body mass index (BMI), homeostatic model assessment for insulin resistance (HOMA-IR), and the inflammatory marker high-sensitivity C-reactive protein (hs-CRP) were significantly associated with aspirin resistance, aligning with previous studies. However, logistic regression analysis did not confirm BMI, hs-CRP, and HOMA-IR as independent risk factors, suggesting they may not individually influence aspirin resistance.

In contrast, multiple linear regression analysis found that hs-CRP and BMI were significant factors affecting the change in urinary 11-dehydro-thromboxane B2 levels after aspirin administration in diabetic patients. This indicates that hs-CRP and obesity, both linked to chronic low-grade inflammation, could collectively impact the aspirin response in diabetes mellitus. The reliance on multiple statistical analyses to support the hypothesis of aspirin resistance in diabetes due to inflammatory pathway activation suggests a complex interplay of factors, making the observation inconclusive, mainly because of the sample size and existence of both ASA sensitive and ASA resistance sub-group within the experimental group. As discussed in the limitation section, metabolic inflammatory markers correlate with aspirin resistance with the existing cohort, their temporal relationship and mechanistic contributions need to be defined.

Finally, more parameters or markers need to be included in the study to assess the inflammation to better understand the ASA resistance in Diabetes patients, which will offer insights for improving cardiovascular disease prevention in this group.

**Response**:Thank you for your insightful feedback. We appreciate your constructive comments and have revised the manuscript accordingly:

1.**Sample Size and Statistical Power:**

We acknowledge the limited sample size as a key constraint affecting the statistical significance of intergroup differences in thromboxane suppression. This limitation is now explicitly addressed in the Limitations section.

2.**COX-2 Upregulation Hypothesis:**

We expanded the Discussion to clarify potential mechanisms, including hyperglycemia-induced COX-2 activation in endothelial cells and accelerated platelet turnover, which may explain residual thromboxane biosynthesis despite ASA therapy(Page [21], Lines [401-403]):”Persistent thromboxane overproduction post-ASA may arise from hyperglycemia-driven COX-2 upregulation in endothelial cells or monocytes—a pathway less sensitive to ASA inhibition[35].”In the Limitations section, we have also included relevant explanations(Page [22], Lines [438-441]):”Moreover, unmeasured inflammatory mediators (e.g., IL-6, TNF-α) and oxidative stress markers (e.g., malondialdehyde) could provide further mechanistic insights into COX-2-driven thromboxane biosynthesis.”

3.**Inconsistent Univariate vs. Multivariable Results:**

We added a discussion on potential collinearity between metabolic-inflammatory markers (e.g., BMI and hs-CRP) and subgroup heterogeneity within the DM cohort, which may explain why these factors were not independent predictors in logistic regression but emerged as significant in linear regression(Page [19], Lines [365-370]):” The discrepancy between univariate and multivariable analyses may reflect collinearity among metabolic-inflammatory variables (e.g., BMI and hs-CRP) or heterogeneous ASA responsiveness within the DM cohort. For instance, subsets with severe insulin resistance or genetic polymorphisms in COX-1/PEAR1 may exhibit distinct pharmacodynamic profiles, diluting the independent effect of individual factors in logistic regression.”

4.**Additional Markers:**

While our study did not measure specific cytokines or oxidative stress markers, we have highlighted the need for future studies to incorporate such parameters in the Limitations and Conclusion sections(Page [19], Lines [365-370]):”Moreover, unmeasured inflammatory mediators (e.g., IL-6, TNF-α) and oxidative stress markers (e.g., malondialdehyde) could provide further mechanistic insights into COX-2-driven thromboxane biosynthesis.”

Thank you again for your valuable input.

Minor remarks

1. Explanation of how ASA resistance is calculated in Table 1 is not provided.

**Response**:Thank you for your valuable feedback. We appreciate your attention to detail. The method for calculating ASA resistance (classification of "good responder" vs. "poor responder") was described in the Methods section under "11dhTxB2 quantification and ASA response assessment" . For clarity, we have now explicitly added the ASA resistance classification criteria to the Table 1 footnote.

Revised Table 1 Footnote:”11dhTxB2(pg/mg creatinine)= [Urinary 11dhTxB2 (pg/mL)] / [Urinary creatinine (mg/mL)];Aspirin resistance (poor ASA responder) was defined as post-administration urinary 11dhTxB2 ≥ 1500 pg/mg creatinine.”

2. The second and third result titles are the same. Used “Univariate analysis” instead of “Multivariate analysis” for the third result title.

**Response**:Thank you for your meticulous review and constructive feedback. We sincerely apologize for the oversight in the original manuscript. As suggested, we have revised the duplicate result titles and corrected the third result section to "**Multivariable analysis of aspirin resistance-related factors in DM patients**" to clearly distinguish it from the univariate analysis. The revised headings now accurately reflect the statistical methods used in each analysis.

Your attention to detail has greatly improved the clarity of our manuscript. Thank you again for your valuable input.

3. Resize the figures for better clarity. Multiple font sizes are used and inconsistent across two figures.

**Response**:Thank you for your valuable feedback on improving the clarity and consistency of the figures. We have carefully revised the figures as per your suggestions.These adjustments enhance visual coherence and align with the manuscript’s professional presentation. Please find the updated figures in the revised submission.Thank you again for your meticulous review.

4. Add significance in figure 1.

**Response**:Thank you for your valuable feedback. We have revised Figure 1 to explicitly indicate the statistical significance of group differences, as suggested:Asterisks (* for baseline comparison, * *for post-intervention) are now displayed above the respective bars in Figure 1.The figure legend has been updated to define these markers: *P =0.001, **P = 0.007 vs. control group.

These revisions enhance the clarity of the statistical outcomes. Please find the updated Figure 1 in the revised manuscript.

Thank you again for your meticulous review.

5. Inconsistency in font used across the manuscript.

**Response**:Thank you for your meticulous attention to formatting details. We sincerely apologize for the inconsistency in font styles across the original manuscript. We have now standardized the font throughout the entire document.Your feedback has significantly improved the professionalism of our manuscript. Thank you again for your valuable input.

We tried our best to improve the manuscript and made some changes in the manuscript. These changes will not influence the content and framework of the paper. And here we did not list the changes but marked in red in revised paper.

We appreciate for Editors/Reviewers’ warm work earnestly, and hope that the correction will meet with approval.

Once again, thank you very much for your comments and suggestions.

Yours sincerely

Bo Chen
