# Supplementary material for: Factors associated with aspirin resistance in diabetic patients: A metabolic and inflammatory profile analysis
Source: PLoS One. 2025 Sep 11;20(9):e0332323. doi: 10.1371/journal.pone.0332323 (PMC12425219; doi:10.1371/journal.pone.0332323)
Supplement: Attachment — Submitted filename: Response_to_Reviewers_auresp_2.docx [file pone.0332323.s004.docx]

Dear Academic Editor :

Thanks for your letter and for the reviewers’ comments concerning our manuscript entitled “Factors Influencing Aspirin Resistance in Diabetic Patients: A Metabolic and Inflammatory Profile Analysis” (ID: PONE-D-25-04526R1). Those comments are all valuable and very helpful for revising and improving our paper, as well as the important guiding significance to our researches. We have studied comments carefully and have made correction which we hope meet with approval. Revised portion are marked in red in the paper. The main corrections in the paper and the responds to the reviewer’s comments are as following:

**The academic editor:**

1. The use of the phrase "factors influencing aspirin resistance" in the title suggests a causal link between the examines variables and the entity referred to as "aspirin resistance." This type of analysis cannot determine causality, it can only investigate an association between the risk variables and the outcome. I would strongly urge the authors to change the title, perhaps to "Factors associated with.." and edit the text to remove any claim of causality.

**Response:** We sincerely thank the academic editor for this crucial and insightful comment regarding the interpretation of our study findings. We fully acknowledge and agree that the observational, cross-sectional nature of our case-control design inherently limits our ability to establish “causality” between the examined metabolic-inflammatory variables and biochemical aspirin resistance. The term "influencing" in the original title could indeed imply a direct causal relationship that our data and study design cannot support. We appreciate the academic editor's constructive suggestion to use more precise language that accurately reflects the associative nature of our findings.

**Revisions made:**

**Title Revision**:The title has been changed to: "Factors associated with aspirin resistance in diabetic patients: A metabolic and inflammatory profile analysis". This revision explicitly frames the study as investigating associations.

**Comprehensive Text Revision**:We have conducted a thorough review of the entire manuscript (Abstract, Introduction, Discussion, and Conclusion) to identify and modify any language that might inadvertently imply causation. Key terms implying causality (e.g., "influence," "promote," "drive," "determine," "effect," "leads to," "contributes to" [when implying direct causation]) have been systematically replaced with terms explicitly denoting association or correlation (e.g., "associated with," "related to," "linked to," "correlates with," "found in," "exhibited by"). We have meticulously ensured that the conclusions drawn strictly reflect the observed “associations” within the limitations of the study design.

**Emphasis on Study Design Limitation**: We have reinforced statements in the Discussion and Limitations sections explicitly stating that our study design identifies associations but cannot establish causation. Specifically, the sentence "Second, the cross-sectional design precludes causal inference..." in the Limitations section now serves as a clearer anchor for this point.

We believe these revisions significantly improve the accuracy and clarity of our manuscript by precisely reflecting the associative nature of our findings, as appropriately constrained by our study design. We are grateful to the academic editor for highlighting this important aspect, which strengthens the scientific rigor of our presentation.

2. The term "aspirin resistance" is often used but I would ask the authors to reflect on and perhaps clarify to what it actually refers. In the broadest sense, it may mean the failure of aspirin to prevent recurrent cardiovascular events. In the narrowest sense, and the one that many experts in the field prefer, it means the failure of aspirin to inhibit platelet thromboxane generation. There is a large body of evidence to suggest that aspirin is very effective at inhibiting platelet thromboxane generation, even in diabetics, and the authors should review these studies. In that sense, "aspirin resistance" defined narrowly is rather rare. The authors use the measurement of thromboxane metabolites in the urine to assess "aspirin resistance." At the time the assay was developed, it was thought that that all thromboxane in the body was produced by platelets and therefore elevated urinary thromboxane metabolites in aspirin users reflected failure of aspirin to inhibit platelet thromboxane generation. Studies that utilized assays reflecting both platelet-specific and total body thromboxane generation using urine metabolite measurement revealed that non-platelet sources of thromboxane generation account for the overwhelming majority of total body thromboxane in compliant aspirin users, even those with diabetes. The reason is thought to be due to the fact that aspirin has a very short half-life (~20 min) and while it inhibits platelet COX-1 for the life of the platelet, nucleated cells can regenerate COX-1 in a few hours after a once daily aspirin dose, or as the authors correctly point out make TXA2 from a COX-2 pathway. The statement that " urinary 11-dehydrothromboxane B2, the primary TXA2 metabolite, directly reflects platelet-derived TXA2 production (line 88) is simply not true. Rather, urine TXA2 metabolites in aspirin non-users reflects both platelet and non-platelet thromboxane generation and in aspirin users reflects predominantly thromboxane generation from non-platelet sources. The truth is, no one really knows how much thromboxane is made from platelet versus non-platelet sources in individuals not on aspirin with systemic diseases such as diabetes. The authors should revise their language to reflect these important nuances.

**Response:** We sincerely thank the academic editor for this exceptionally insightful and critical comment regarding the precise definition of "aspirin resistance" and the interpretation of urinary 11dhTxB2 levels, particularly in the context of non-platelet sources. We appreciate the opportunity to clarify these crucial nuances, which significantly strengthen the scientific accuracy and interpretation of our findings.

**Actions Taken in Response:**

**Clarified Definition of Aspirin Resistance**: We have explicitly defined the specific type of "aspirin resistance" investigated in our study as "biochemical aspirin resistance" throughout the manuscript (Abstract, Methods, Results, Discussion, Conclusion). We emphasize that this definition is based “solely” on persistent thromboxane biosynthesis (urinary 11dhTxB2 ≥ 1500 pg/mg creatinine post-ASA) and “does not imply clinical treatment failure” (i.e., recurrent cardiovascular events). We have added text in the Introduction and Methods sections explicitly stating this distinction and referencing the ongoing debate regarding its clinical relevance. We also acknowledge that true failure of aspirin to inhibit “platelet-specific” COX-1 is likely rare.

**Revised Interpretation of Urinary 11dhTxB2:** We have completely revised statements regarding the source specificity of urinary 11dhTxB2. Crucially, we have removed the claim that it "directly reflects platelet-derived TxA2 production" (original line 88). We now accurately state that urinary 11dhTxB2 reflects “total body TxA2 biosynthesis”. We explicitly acknowledge that in aspirin-treated individuals, the “majority” of urinary 11dhTxB2 likely originates from “non-platelet sources” (e.g., endothelial cells, monocytes via COX-2 or regenerated COX-1), especially given aspirin's short systemic half-life and the ability of nucleated cells to resynthesize COX. This revision is incorporated into the Introduction (where the biomarker is first described) and extensively discussed in the context of our findings.

**Incorporated Discussion of Non-Platelet Sources:** We have significantly expanded the Discussion section to address the critical point raised by the academic editor regarding non-platelet sources of TxA2 biosynthesis. We discuss:

The evidence that in aspirin-treated individuals (including diabetics), urinary TxA2 metabolites predominantly reflect non-platelet sources.

The potential mechanisms driving non-platelet TxA2 production in diabetes (e.g., hyperglycemia-induced COX-2 upregulation in endothelial/monocytic cells).

How this understanding contextualizes our finding of persistent 11dhTxB2 elevation post-ASA in diabetics, suggesting it may reflect “systemic (potentially COX-2 mediated) thromboxane overproduction” rather than (or in addition to) true platelet COX-1 resistance.

That the relative contribution of platelet vs. non-platelet sources to baseline (pre-ASA) urinary 11dhTxB2 in individuals with systemic diseases like diabetes remains

**Nuanced Language Throughout:**We have carefully revised language related to 11dhTxB2 and aspirin resistance across the entire manuscript (Abstract, Introduction, Methods, Results, Discussion) to incorporate these nuances, replacing definitive claims about platelet specificity with accurate descriptions of total body TxA2 biosynthesis and the complexity of its sources, particularly under aspirin therapy.

**Re-evaluation of "Resistance" Terminology:**While we retain the term "biochemical aspirin resistance" for consistency with established literature using this urinary metabolite threshold, we now explicitly frame it within its limitations: it indicates “inadequate suppression of ‘total body’ TxA2 biosynthesis” by the administered aspirin regimen, the source of which is likely predominantly extra-platelet. We discuss the potential clinical implications (association with cardiovascular risk) despite this lack of source specificity.

We are deeply grateful to the academic editor for highlighting these fundamental aspects of thromboxane biology and aspirin pharmacology. Their critique has led to substantial improvements in the accuracy, depth, and scholarly rigor of our manuscript.

3.The authors did not measure and consider oxidative stress as a variable in their analysis. While they touch on this briefly under Limitations, they should expanding this discussion of this limitation. Oxidative stress has been shown in multiple studies by several groups to be one of the strongest risk factors for thromboxane generation. For example, in a study of aspirin users with documented complete suppression of platelet TXA2 generation, our group found oxidative stress was the most important variable contributing to elevated urinary TXA2 metabolites (i.e. non-platelet TXA2 generation) that was directly associated with increased thrombosis and mortality (PMID 27068626, 29097390). Similar results were found in the largest study to date to investigate the association of urinary TXA2 metabolites and outcome (involving over 3000 Framingham participants), where oxidative stress was a stronger risk factor for elevated urinary thromboxane levels than diabetes and inflammation (PMID 35660296).

**Response:** We sincerely thank the academic editor for this critically important insight and for highlighting a significant limitation in our study design. We fully acknowledge that oxidative stress is a pivotal driver of thromboxane biosynthesis, particularly from non-platelet sources, and its omission as a measured variable represents a substantial gap in our analysis. We appreciate the academic editor's guidance in directing us to seminal studies, including their own foundational work and the large-scale Framingham cohort analysis, which robustly demonstrate that oxidative stress is a stronger predictor of thromboxane overproduction than traditional risk factors like diabetes or inflammation.

In response to this valuable critique, we have substantially expanded the discussion of this limitation in the manuscript. Specifically, we have:

Added detailed text in the Limitations section explicitly acknowledging oxidative stress as an unmeasured confounder and discussing its established mechanistic role in driving non-platelet thromboxane generation.

Enhanced the Discussion section to contextualize how oxidative stress pathways may underlie our observed associations between metabolic-inflammatory markers (hs-CRP, HOMA-IR) and persistent 11dhTxB2 elevation.

Cited the recommended references (PMID 27068626, 29097390, 35660296) to support these points.

Proposed specific oxidative stress biomarkers and targeted therapeutic strategies in the Future Directions section.

These revisions significantly strengthen the manuscript by providing a more comprehensive perspective on the determinants of thromboxane biosynthesis and aspirin response variability. We are deeply grateful to the academic editor for this expert guidance, which has greatly enhanced the scholarly rigor of our work.

4. The authors should be aware that the 1500 pg/mg creatinine threshold for determining aspirin responsiveness was derived from the manufacturer of the assay and not based on any outcome analysis but rather on comparison to results from a first-generation assay whose threshold was determined arbitrarily (see PMID 38416712 for an outcome analysis of different thresholds for this assay).

**Response:** We sincerely thank the academic editor for highlighting this important methodological consideration regarding the 1500 pg/mg creatinine threshold for defining biochemical aspirin resistance. We acknowledge that this threshold was primarily derived from the manufacturer's assay validation data and lacks robust outcome-based clinical validation. As appropriately noted, recent evidence (PMID 38416712) demonstrates significant variability in outcome associations across different thresholds for this assay.

To address this limitation, we have:

Revised the Methods section (11dhTxB2 Quantification subsection) to explicitly state the origin of the threshold and its limitation regarding clinical outcome correlation.

Added the suggested reference (PMID 38416712) to support this clarification.

These revisions enhance the transparency of our methodology and appropriately contextualize the interpretation of "biochemical aspirin resistance" prevalence. We believe these changes strengthen the manuscript without altering our core findings.

We tried our best to improve the manuscript and made some changes in the manuscript. These changes will not influence the content and framework of the paper. And here we did not list the changes but marked in red in revised paper.

We appreciate for Editors’ warm work earnestly, and hope that the correction will meet with approval.

Once again, thank you very much for your comments and suggestions.

Yours sincerely

Bo Chen
